# Innovative Seismic Solutions for Precast Structures: Experimental and Numerical Studies on Beam–Column Joints

**DOI:** 10.3390/ma18215049

**Published:** 2025-11-06

**Authors:** Roberto Nascimbene, Davide Bellotti

**Affiliations:** 1 Department STS, IUSS—Scuola Universitaria Superiore Pavia, 27100 Pavia, Italy; 2 EUCENTRE—European Centre for Training and Research in Earthquake Engineering, 27100 Pavia, Italy; davide.bellotti@eucentre.it

**Keywords:** seismic resilience, precast frame systems, beam-to-column connections, energy dissipation, performance-based design, dry construction methods

## Abstract

This study presents a novel structural framing solution designed to improve seismic energy dissipation and limit displacements, aiming to serve as an effective alternative to traditional precast systems employing pendulum-based isolation. While pendulum mechanisms mitigate seismic forces by decoupling the superstructure from ground motion, they are typically characterized by high implementation costs, mechanical complexity, and post-event maintenance challenges. In contrast, the proposed approach integrates seismic performance enhancements within the structural frame itself, removing the dependency on external isolation components. The system leverages a combination of pinned and semi-rigid beam-to-column joints that are tailored for use within dry precast construction technologies. These connection types not only support rapid and labor-efficient assembly but also, when properly detailed, offer robust hysteretic behavior and deformation control under dynamic loading. The research includes both experimental testing and numerical simulations focused on the cyclic response of these connections, enabling a comprehensive understanding of their role in dissipating energy and delaying damage progression. Recognizing the industry’s frequent emphasis on construction speed and upfront cost-efficiency, often at the cost of long-term reparability, this work introduces an alternative framework that emphasizes resilience without compromising construction practicality. The resulting system demonstrates improved post-earthquake functionality and reduced downtime, making it a promising and economically viable option for seismic applications in precast construction. This advancement supports current trends toward performance-based design and enhances the structural reliability of dry-assembled systems in seismic regions.

## 1. Introduction

In the European construction context, precast concrete buildings, particularly in the industrial sector, are frequently characterized by notable structural flexibility [1,2,3,4,5]. This flexibility stems largely from the prevalent use of slender cantilever columns [6,7,8] and beam-to-column connections designed as pinned or exhibiting limited rotational stiffness [9,10,11]. These joints typically depend on frictional mechanisms or proprietary mechanical inserts, which, despite their simplicity, have demonstrated poor performance under seismic loading [12,13,14]. Notably, field observations following recent Italian earthquakes have revealed multiple instances of connection failure, leading to beam dislodgement and, in severe cases, complete loss of vertical support [15,16,17]. Such failures raise critical questions about the seismic reliability of existing precast systems.

These vulnerabilities have drawn attention to the urgent need for broad-based assessment and retrofitting strategies [18,19,20,21] targeting aging precast industrial buildings across Europe. Given their role in supporting economic and logistical infrastructures, ensuring the seismic safety of these facilities has become a high priority. A crucial preliminary step involves developing a detailed taxonomy of structural configurations tailored to national or regional construction practices, an effort increasingly reflected in the literature [22]. This classification enables targeted vulnerability assessments and informs retrofitting strategies aligned with prevalent construction patterns [23,24,25].

Typical industrial precast buildings in Southern and Central Europe consist of single-story, multi-bay frames, the main span of which is oriented transversely [21,26,27]. Slender precast columns in socket foundations support long prestressed beams (14–20 m). Beam-to-column joints are often idealized as pinned, though semi-rigid in reality, leading to underestimated seismic response. Longitudinal members rarely contribute to lateral resistance, making overall behavior dependent on column flexure and roof diaphragm action. Seismic vulnerability increases due to heavy non-structural components [28,29,30,31,32,33,34,35,36,37,38,39,40,41,42,43], such as equipment, piping, and cladding, which add mass, lengthen the fundamental period, and amplify displacement demands.

A substantial fraction of Italy’s precast industrial stock was built during a period when many municipalities were not yet classified as seismic zones [44,45]. As a result, buildings erected over the past 60 years often lack proper seismic detailing or reinforcement anchorage, despite their widespread use. Earthquake sequences in recent decades, including the 2009 L’Aquila event [46,47,48], the 2012 Emilia-Romagna earthquakes [12,13,14,15,49], and the 2016–2017 Central Italy series [50,51,52], have collectively revealed the extent of structural vulnerability in this typology [53]. Similar patterns of vulnerability have been documented globally in other seismic-prone regions, including Turkey [54,55], New Zealand [56,57], and beyond [58,59], where precast construction typologies exhibit comparable weaknesses. These failures are consistently attributed to the lack of energy dissipation mechanisms, discontinuous or poorly detailed structural connections, and an excessive degree of flexibility. The convergence of these factors leads to inadequate seismic performance, particularly when dynamic demands exceed the limited deformation and ductility capacities of such systems.

In light of these challenges, this study proposes a reimagined frame configuration capable of delivering superior energy dissipation and displacement control under seismic loads, offering an alternative to conventional pendulum-type isolation systems. The proposal is grounded in extensive analytical and numerical studies carried out in recent years [60,61,62,63,64]. The proposed system has been evaluated against multiple performance criteria, including structural effectiveness, constructability, economic impact, and reparability following seismic events.

Crucially, the proposed frame adheres to the construction logic familiar to the precast industry. It makes use of a hybrid connection philosophy that combines hinged joints and semi-rigid (partially restrained) interfaces, delivering enhanced seismic resilience without introducing executional complexity. The solution maintains compatibility with dry-assembly methods, a defining feature of modern precast construction, thereby supporting efficient installation, minimal on-site labor, and ease of maintenance. By bridging performance-based seismic demands with practical construction workflows, the system provides a promising pathway for upgrading the resilience of European precast building stock.

While several previous European projects have explored hybrid or semi-rigid precast joints, the present study introduces a novel configuration that combines dry-assembly technology, replaceable dissipative devices, and reversible steel connectors into a single system. This integration allows both stable energy dissipation under cyclic loading and complete demountability, enabling structural reuse and rapid post-earthquake repair. The originality of this work, therefore, lies in the mechanical detailing, functional hybridization, and circular-design integration of the joint, rather than in the general concept of hybrid connections.

## 2. Structural Configuration and Detailing of Beam-to-Column Joints

**Figure 1:**
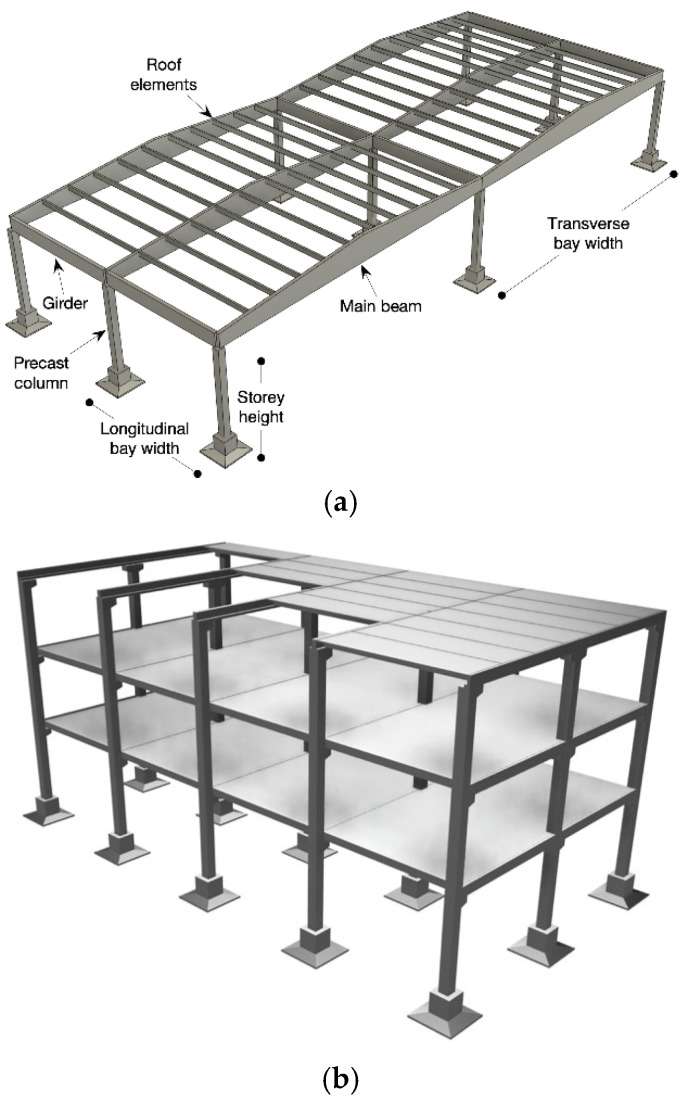
Common precast typologies in European industrial buildings: (**a**) single-storey precast and (**b**) multi-storey skeleton bearing frame structural typologies.

The predominant structural typologies found within the European, and particularly Italian, precast reinforced concrete industrial building stock [22,26] are schematically illustrated in Figure 1, which provides a qualitative overview of the reference cases considered in this study. Among these, the most common configuration consists of a series of single-storey portal frames arranged along the transverse direction (Figure 1a). Each frame typically comprises two or more columns rigidly embedded or socketed at the base [61], supporting a prestressed concrete main beam, often with a double-pitched profile. These beams are connected to the columns through dry frictional interfaces or dowel-based systems [65,66], lacking dedicated energy dissipation mechanisms [67].

The roofing system in such single-storey buildings generally consists of longitudinally spanning prestressed double-tee elements, which are rigidly fastened to the transverse main beams, contributing to diaphragm action in the horizontal plane. A structurally analogous yet vertically extended variant of this configuration is represented by multi-storey precast buildings (Figure 1b). These are typically composed of monolithic vertical elements and pin-ended main beams resting on corbels. The adoption of pinned connections at beam ends inherently precludes the development of dissipative zones within the horizontal framing system, concentrating inelastic demands, if any, at the column bases.

Main beams in these multi-storey configurations are generally L-shaped along the perimeter frames and inverted T-shaped in internal bays, reflecting standard precast manufacturing practices. Secondary beams may also be present, typically seated on corbels and likewise pinned. Horizontal diaphragms at intermediate levels are formed by prestressed hollow-core slabs, while roof systems often utilize the same double-tee units employed in single-storey buildings. This typological continuity across different building heights underlines the widespread and standardized nature of dry precast construction in the region.

**Figure 2:**
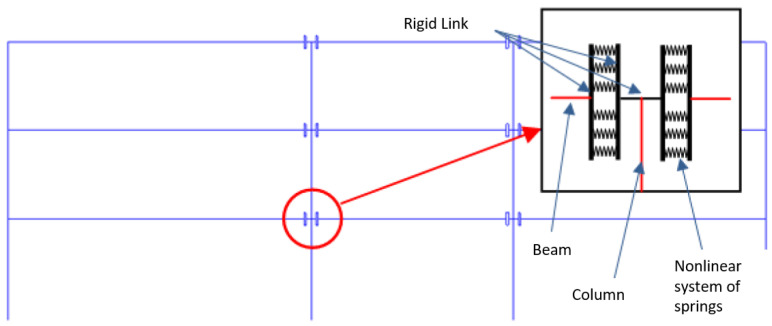
Schematic representation of the proposed three-span precast frame configuration, featuring hinged connections at the external columns and partially restrained (semi-rigid) joints at the internal columns. This hybrid connection strategy enhances global stiffness and stability while maintaining compatibility with dry construction methods.

Building upon the analysis of prevalent structural typologies, a representative case study was developed to isolate and investigate the beam-to-column connection subsystem through both numerical simulation and experimental testing. The selected structural configuration, Figure 2, features a three-span frame with hinged connections at the outer columns and partially restrained joints at the internal supports (to be tested in the next sections). This hybrid setup provides a significant improvement in overall lateral stiffness and offers better control over second-order (P−Δ) effects and interstorey drifts, particularly at the damage limitation state. Compared to conventional fully pinned systems, this approach enables the use of smaller column cross-sections, optimizing material efficiency and construction simplicity. Additionally, the layout is inherently compatible with the integration of supplemental damping devices, such as friction or viscous dissipators [20,68], positioned at the hinged ends to further enhance seismic energy dissipation.

To examine the structural behavior of this system, a detailed case study was performed on a three-storey frame with span lengths of 12 m, 10 m, and 8 m, and interstorey heights of 4.0 m at ground level and 3.5 m at the upper floors. The model was developed using MIDAS GEN ver. 2025 [69], incorporating nonlinear beam elements with distributed plasticity to capture flexural behavior [70].

Given the nature of partial fixity at internal joints, the beam-to-column interface was modeled via a combination of vertical rigid links and parallel nonlinear springs (Figure 2), simulating the collective response of grouted socketed connections and embedded threaded bars. A unilateral constraint was introduced at the corbel to reflect realistic compression-only support and to capture the nonlinear flexural interaction. The frame was designed for a site in Gemona del Friuli (Udine Province, Northern Italy), characterized by soil type C and a peak ground acceleration (PGA) of 0.25 g according to local seismic zoning.

**Figure 3:**
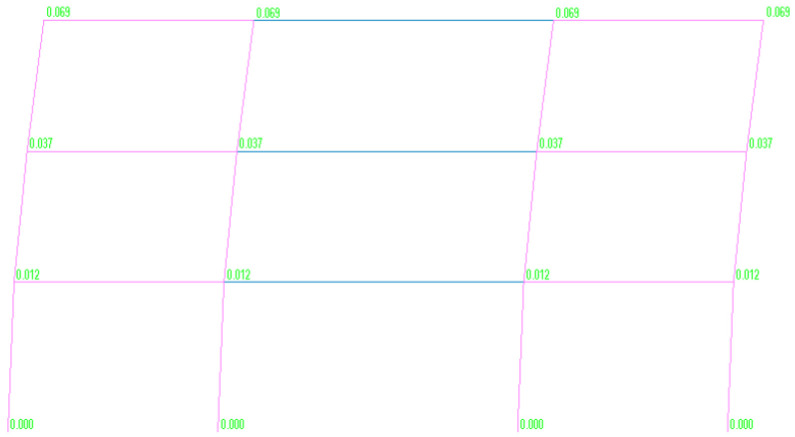
Displacement demand at each storey level obtained from the nonlinear analysis in MIDAS GEN for the proposed three-span precast frame configuration. The model features hinged connections at the external columns and partially restrained (semi-rigid) joints at the internal columns, allowing improved control of lateral displacements under seismic loading.

The initial design included column dimensions of 70 × 50 cm at the ground floor and 50 × 50 cm at the upper levels. However, P−Δ stability checks under seismic loads revealed that the second and third-storey columns failed to meet the required stability criteria, while the ground-level columns required enhanced resistance verification. Since the equilibrium conditions necessitated the application of a uniform amplification factor (θ) across all storeys, according to the NTC 2018 code [71] and also Eurocode [72], a global redesign of the column system was necessary. The final configuration, ensuring compliance with relevant code provisions [22], adopted uniform column dimensions of 80 × 80 cm across all levels. Figure 3 illustrates the deformed shape of the three-span frame incorporating the redesigned column dimensions, as obtained from the nonlinear analysis. The corresponding interstorey drifts at each level, ground, first, and second storey, are reported in Table 1, providing a quantitative assessment of lateral displacement demands. Additionally, Table 2 summarizes the results of the verification carried out for second-order effects (P−Δ), highlighting the influence of the updated column dimensions on the overall stability and compliance with design code requirements.

Following the definition and modeling of the global frame system, the study shifts focus to the characterization and analysis of its most critical component: the beam-to-column connection subsystem. A central innovation of the proposed structural solution lies in the use of partially restrained joints, specifically engineered to enhance both lateral stiffness and energy dissipation under seismic loading.

The connection mechanism is based on a grouted sleeve anchorage system, in which threaded steel bars protrude from the precast beam and are inserted into preformed cavities within the column. These cavities, typically less than or equal to 10 cm in depth, are filled with high-strength cementitious grout to ensure effective force transfer. The threaded bars are designed primarily to carry axial loads, thereby maximizing the efficiency of the connection in resisting tension and compression during seismic excitation.

To mitigate the risk of grout cracking due to localized tensile stresses, each bar is encased in a 3 mm thick rubber sheath along its embedded length. This detail reduces mechanical interlock between the bar and the grout, thereby limiting stress concentrations and accommodating small deformations without inducing damage in the grout interface.

The anchorage strategy is tailored according to the joint location. In internal cruciform joints, continuity is achieved either through direct overlapping of reinforcement bars or via mechanical couplers. In external T-joint configurations, the bars are anchored using bearing washers or bonded directly to the surrounding concrete mass.

Vertical support of the beam is provided by a standard reinforced concrete corbel, incorporating a 1 cm neoprene pad to accommodate vertical displacements and reduce localized stress concentrations. The corbel is dimensioned to resist shear forces in only one direction. In the event of seismic load reversals, the design deliberately transfers uplift shear forces to the reinforced beam section itself, rather than relying on frictional or corbel resistance. This conservative approach ensures robust performance under dynamic conditions by minimizing dependence on uncertain frictional behavior and enhancing the overall reliability of the connection.

## 3. Advanced Local Finite Element Model

**Figure 4:**
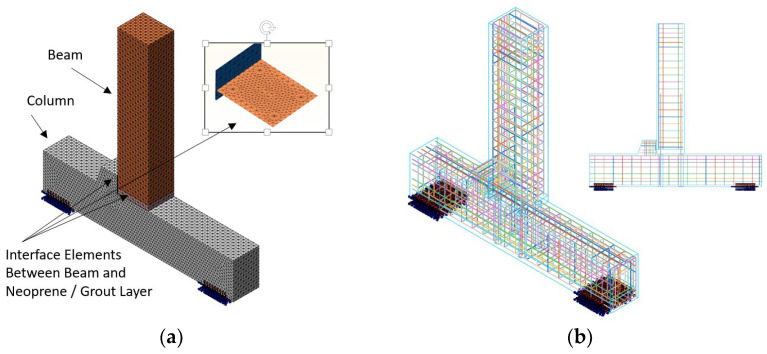

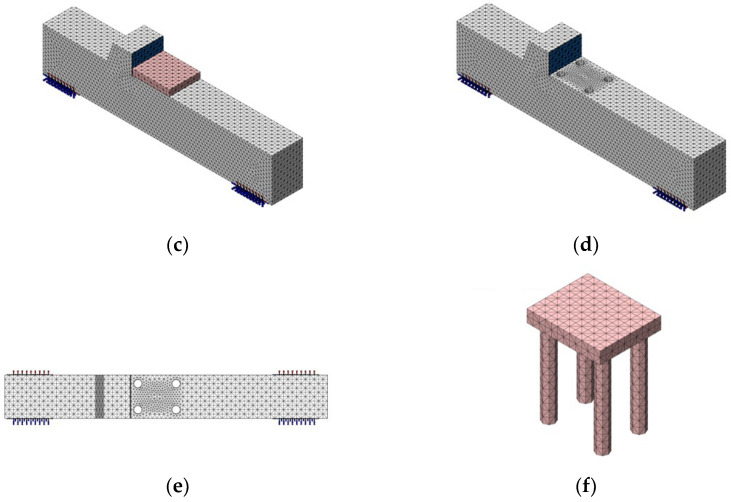
(**a**) Finite element model of the beam-to-column joint developed in MIDAS FEA; (**b**) detail of embedded reinforcement within the beam and column elements; (**c**) column with grout layer; (**d**) column only, with visible anchorage holes; (**e**) top view of the column; (**f**) grout layer component.

The local numerical modeling (Figure 4a) of the beam-to-column joint represents a fundamental step in bridging the global structural case study described in Section 2 with the detailed design and calibration of the experimental campaign presented in Section 4. This modeling effort was carried out using MIDAS FEA ver. 2025 [73], leveraging solid finite elements to capture the nonlinear mechanical behavior of the various components involved. In particular, solid elements were employed to represent the concrete of both the beam and the column, as well as the cementitious grout, which plays a crucial role in both the horizontal connection layer and in the grouted sleeves anchoring the threaded bars into the column body. The reinforcement system (Figure 4b) was modeled with a hybrid approach [74]. Internal bars, fully embedded within the concrete cross-section, were defined using “embedded” elements to ensure bond compatibility with the surrounding matrix. In contrast, through-bars crossing the grout layer were modeled using truss elements, carefully calibrated to reflect their axial stiffness and transfer capacity across the connection zone. This approach enabled a more accurate representation of the load path through the joint, particularly under cyclic or reversing seismic demands.

To simulate the potential interface behavior [74] between different materials, interface elements, similar in formulation to contact elements, were introduced (Figure 4c–f). These elements allowed for the simulation of separation and slip phenomena between the beam and the neoprene pad [61,74], as well as between the beam and the underlying grout layer, both of which are critical for capturing localized failure mechanisms and stress redistribution within the joint.

The constitutive laws assigned to each material were chosen to reflect their distinct nonlinear behavior. For both the concrete and the cementitious grout, the Thorenfeldt model was adopted [75] in compression, capturing the strain-softening behavior beyond peak stress ($\varepsilon_{c1}$ = 0.0020 and ultimate strain $\varepsilon_{cu}$ = 0.0035). In tension, an exponential softening law was implemented to simulate crack initiation and propagation ($f_{t}$ ≈ 3.5 MPa and fracture energy $G_{f}$ = 0.10 N/mm). The steel reinforcement was modeled using a multi-linear stress–strain curve, allowing for plastic deformation and post-yield behavior to be captured with adequate accuracy. A parametric series of simulations was conducted, considering different hypotheses regarding two critical aspects: (i) the tensile strength of the grout–concrete interface, which governs the onset of cracking between the beam and the connection layer, and (ii) the bond–slip behavior of the bars crossing this cracked region, which strongly influences energy dissipation and stiffness degradation under cyclic loads.

The grout–concrete interface was modeled with a bilinear cohesive traction–separation law (mode-I and mode-II) characterized by elastic stiffnesses $K_{n}$ and $K_{s}$, peak strengths $\sigma_{n,\max}$ and $\tau_{\max}$, and fracture energies $G_{nc}$ and $G_{sc}$. Stiffnesses were derived from the equivalent modulus of grout/concrete and the measured interface thickness h via $K_{n}\;\approx E_{\mathrm{eq}}/h$ and $K_{s}\;\approx G_{\mathrm{eq}}/h$ (with h = 3–5 mm). Peak tensile strength was taken as $\sigma_{n,\max}=\alpha f_{t,\mathrm{grout}}$ with α = 0.5 ± 0.1, where $f_{t,\mathrm{grout}}$ was inferred from the Emaco S55 compressive strength using standard correlations; the shear strength followed $\tau_{\max}=c_{0}+\mu \sigma_{n}$ with $c_{0}\;=$ 0.5–0.8 MPa and μ = 0.6. Fracture energies adopted $G_{nc}\;$= 0.10 ± 0.04 N/mm and $G_{sc}$ = 0.15 ± 0.05 N/mm, consistent with published ranges for cementitious interfaces. Beam–neoprene and column–neoprene contacts used compression-only hard contact with Coulomb friction μ = 0.20–0.30. Normal penalties were selected to keep numerical penetration < 0.5% of pad thickness while avoiding ill-conditioning.

At the current modeling stage, the phenomenon of partial flexure or buckling of the threaded bars, which was observed during subsequent experimental tests, has not yet been explicitly incorporated. As a result, the numerical predictions tend to be conservative, providing a lower-bound estimate of the joint’s real-world performance. Nevertheless, it is anticipated that by introducing a degradation mechanism, for example, through a calibrated reduction in the post-yield branch of the steel constitutive model, the numerical results could be further refined, achieving closer agreement with experimentally observed behavior.

**Figure 5:**
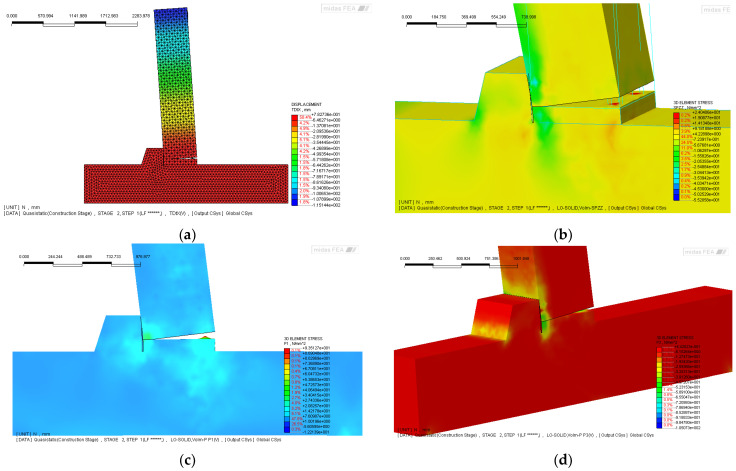

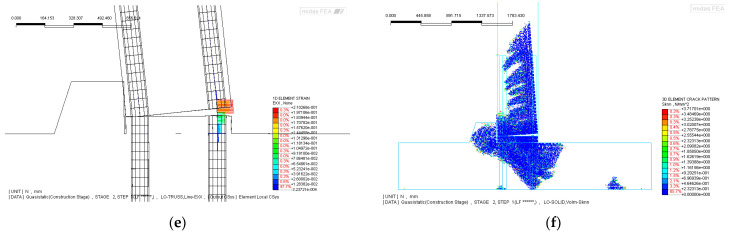
Finite element results of the beam-to-column joint: (**a**) deformed shape with interface elements and reinforcement layout; (**b**) vertical stress ($\sigma_{zz}$) showing load transfer mainly through the beam; (**c**,**d**) principal stress fields ($\sigma_{P1}$ and $\sigma_{P3}$) indicating tension zones and potential cracking paths; (**e**,**f**) axial forces in truss elements and crack pattern in the beam under seismic loading.

The results of the finite element simulations are presented in Figure 5, which illustrates the deformed configuration and the corresponding stress distributions within the joint model. A key feature emerging from the analysis is the detachment between the column face and the grout layer, alongside the formation of a diagonal strut mechanism linking the beam to the column via the corbel. These stress paths are particularly relevant in interpreting the seismic behavior of the connection.

Figure 5a shows the overall deformed shape of the numerical model, developed in MIDAS FEA [73], which includes interface elements between beam and grout, beam and neoprene, and column and neoprene. The reinforcement is modeled using a combination of “embedded” bars and truss elements representing the bars crossing the grout interface. This detailed modeling strategy allows for a realistic simulation of both the bonded and unbonded portions of the connection, capturing the complex interactions under seismic loading. The vertical stress distribution ($\sigma_{zz}$), shown in Figure 5b, reveals that the majority of the vertical load is carried by the beam itself, while the corbel remains practically unstressed. This finding is highly significant: as discussed in the introduction and supported by several experimental [34,36,39,40,61,70] and post-earthquake field investigations [12,13,14,15,17,44], brittle failure at the corbel edge, particularly due to concrete spalling or crushing, has often led to a critical reduction in the effective bearing length. This, in turn, results in loss of support and potential collapse of the structural system, a recurring vulnerability in precast industrial buildings affected by seismic events. Figure 5c,d further support this interpretation, showing the principal stress fields ($\sigma_{P1}$ and $\sigma_{P3}$) in the joint region. The orientation and magnitude of the principal stresses confirm the development of tension zones aligned with potential crack paths, especially in the interface area and along the underside of the beam. These stress concentrations help explain the early onset of damage observed in similar connection configurations.

Finally, Figure 5e,f focus on the truss elements representing the bars passing through the grout interface, highlighting their stress state and the crack propagation behavior in the beam. The simulation results clearly show that significant tensile strains develop in these bars, indicating progressive bond degradation and crack widening. These observations are consistent with the hypothesis of partial flexure or yielding of the bars, an effect that, although not explicitly included in the current model, was later observed during experimental testing. Taken together, the numerical results provide strong evidence of the joint’s critical behavior under seismic loading and underscore the importance of conservative detailing to ensure robustness and prevent brittle failure modes.

## 4. Experimental Setup and Assembly Procedure of the Beam-to-Column Subassembly

**Figure 6:**
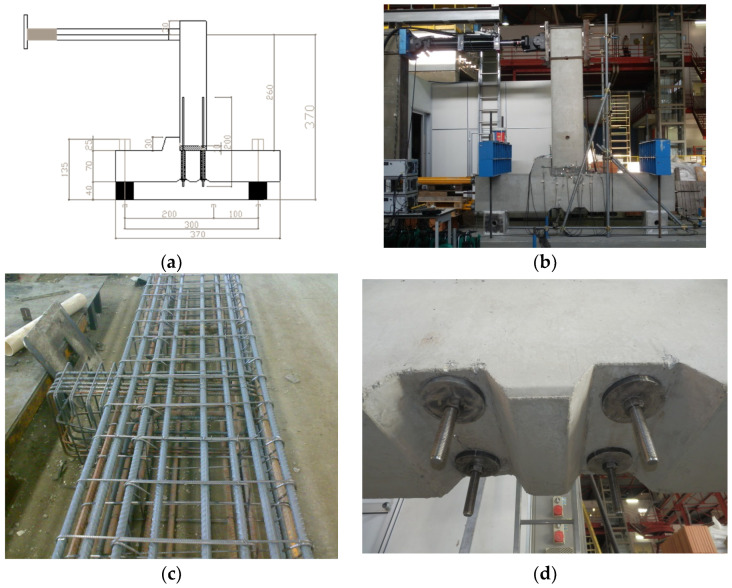
Experimental setup of the beam-to-column subassembly: (**a**) schematic representation of the test specimen, including beam, column, and corbel; (**b**) laboratory positioning of the assembled specimen with horizontal column and vertical beam; (**c**) detail of reinforcement layout between corbel and beam; (**d**) external anchorage system for the threaded bars, consisting of 10 mm-thick, 150 mm-diameter circular steel plates and high-strength bolts.

The beam-to-column subassembly (Figure 6a,b) selected for experimental testing was extracted from the reference structural typologies previously modeled in MIDAS GEN [69] (Section 2) and calibrated through the local finite element model described earlier (Section 3). The connection to be tested incorporates an external anchorage system, consisting of steel end plates and bolts, housed within two grooves embedded in the column section, specifically designed to accommodate the post-installed threaded bars (Figure 6d). The physical specimen comprises a 3.7 m long column segment and a 2.7 m long beam segment, both representative of typical precast elements.

A reinforced concrete corbel (Figure 6c) is integrated into the column to replicate the beam support condition, with a 10 mm neoprene pad placed between the beam and the corbel to allow for local stress redistribution and accommodate potential vertical displacements. The cross-section of the column is 70 × 50 cm, while the beam cross-section is 60 × 50 cm, consistent with the structural design of a three-storey, three-span precast frame, from which this joint configuration was originally derived.

In the laboratory, the specimen was assembled with the column placed horizontally and the beam oriented vertically (Figure 6b). This configuration was selected for practical reasons, mainly to facilitate the positioning of the actuator and load application. The column was supported by two 40 cm high concrete blocks, allowing sufficient space for joint deformation. Anchorage to the laboratory strong floor was achieved using two sets of post-tensioned steel beams, anchored with Dywidag bars, ensuring adequate restraint against uplift and sliding. The beam–column subassembly was tested with the beam in a vertical orientation and the column horizontal. This configuration was adopted for practical reasons, enabling precise control of cyclic loading, improved instrumentation accessibility, and stable boundary constraints. Although the geometric orientation differs from that of in situ assemblies, the imposed loading reproduced the same internal force path and flexural–shear interaction as those experienced by the connection in a real frame under horizontal seismic excitation. The constant axial load applied to the column simulated gravity effects, maintaining the realistic stress ratio between flexural and axial actions. Such a setup is consistent with experimental practices adopted in previous studies on precast joints and ensures adequate representativeness of the joint behavior under cyclic conditions.

The reinforcement layout of the column and beam segments was designed based on the internal reinforcement specified in the original frame model. The material properties used for the test specimen were as follows:

- Concrete (columns and beams): C40/50;
- High-strength grout: Emaco S55;
- Reinforcement steel: B450C;
- Threaded bars (anchorage system): Grade 8.8.

The assembly process began with the positioning of the column on the concrete supports. The contact surfaces were treated with a layer of high-strength grout to improve friction and adhesion. The beam was then lowered from above, temporarily elevated 10 cm above the column face using wooden wedges. Vertical alignment was achieved through the external steel endplate and bolt system.

Once the beam was correctly positioned, side formwork was installed, leaving one face open (the one opposite the corbel) to allow casting of the grout infill layer (Emaco S55). Special attention was paid to ensuring complete grout penetration into the four corrugated ducts embedded in the column, used for bar anchorage. The external plates remained untightened during casting, allowing grout to escape from the bottom outlet, confirming full cavity filling and eliminating air pockets. The plates were tightened only after the grout had fully filled the anchorage ducts.

It is worth noting that this laboratory procedure is more complex than the on-site counterpart, where the beam would typically be installed horizontally, and the grout could be poured from above directly into the connection cavity, facilitating full filling by gravity. An important consideration concerns the cost and performance of the grout used for the connection. Emaco S55, while offering excellent mechanical properties and fluidity, is relatively expensive—approximately €8 per 10 kg, VAT included. Each tested connection required between 130 and 140 kg of grout, resulting in a material cost of around €110 per specimen. While suitable for experimental validation and high-performance applications, future studies may explore more economically sustainable alternatives for large-scale implementation in real structures.

The experimental setup was extensively instrumented to capture the global and local response of the beam-to-column subassembly during testing. A total of 29 displacement transducers (LVDTs) were installed on the specimen to monitor relative displacements, rotations, and potential detachment phenomena at critical interfaces. Eight vertical LVDTs were mounted at the base of the column on the faces perpendicular to the loading direction, four with a 25 mm stroke and four with a 50 mm stroke, to measure vertical movements and potential uplift. An additional group of four horizontal LVDTs was positioned on the faces parallel to the loading axis to record the slip between the grout layer and the foundation, as well as between the column and the foundation. A similar set of four vertical LVDTs, placed on the same faces, was used to detect any detachment of the grout layer or the column from the foundation surface. To characterize the global response of the system, four horizontal LVDTs with extended measurement ranges were installed along the height of the column: one at the top (250 mm stroke), one at mid-height (250 mm stroke), one near the base (125 mm stroke), and one at the foundation level (25 mm stroke), allowing for precise determination of lateral displacements and overall drift. Two additional vertical LVDTs (25 mm stroke) were installed on the foundation to record its vertical settlement during cyclic loading. Finally, six more sensors were distributed on the faces parallel to the loading direction, four vertical and two horizontal, each with a 50 mm stroke, to provide redundant measurements and to verify symmetry and consistency of the deformation field. This comprehensive instrumentation layout enabled accurate monitoring of both local interaction effects at the joint interfaces and the global deformation pattern of the entire subassembly throughout the test.

**Figure 7:**
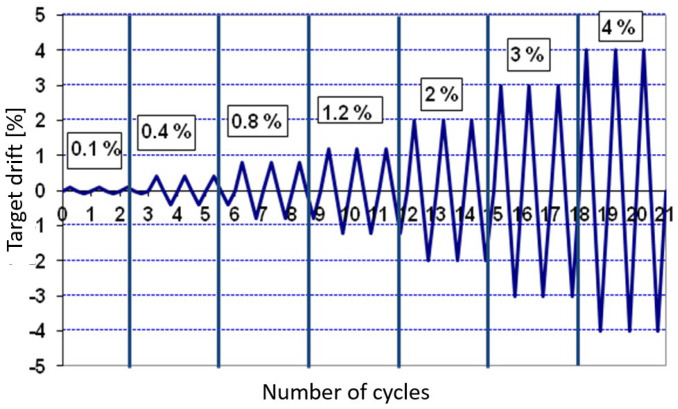
Displacement time history applied to the beam end during the quasi-static cyclic loading protocol, used to simulate increasing drift demand under seismic conditions.

A quasi-static cyclic displacement history was applied to the top end of the beam, with increasing drift levels, to simulate seismic loading conditions. The complete displacement protocol is summarized in Table 3 and graphically illustrated in Figure 7. To replicate the axial load typically acting on a column in real structures, the column was post-tensioned prior to testing. This setup ensured a realistic boundary condition and allowed for the evaluation of the joint’s performance under combined axial and lateral loading conditions.

The cyclic test was conducted under displacement-controlled loading using symmetric cycles of increasing amplitude applied quasi-statically at the beam tip. The loading sequence followed a modified FEMA 461 protocol, allowing precise control of cyclic drifts and consistent evaluation of hysteretic behavior. A constant axial load equal to approximately 10% of the column’s axial capacity was maintained throughout the test to represent gravity effects. This ensured that the measured energy dissipation and stiffness degradation were governed by lateral deformation mechanisms rather than by variations in axial load.

## 5. Experimental Results

As anticipated based on the local finite element modeling (Section 3), the experimental results confirmed the formation of a progressive detachment between the beam and the grout layer, marking a transition from a nominally fixed connection to a behavior more accurately described as semi-rigid. This detachment became distinctly visible at an imposed drift of approximately 0.8%, coinciding with the onset of micro-cracking and degradation in the grout interface.

**Figure 8:**
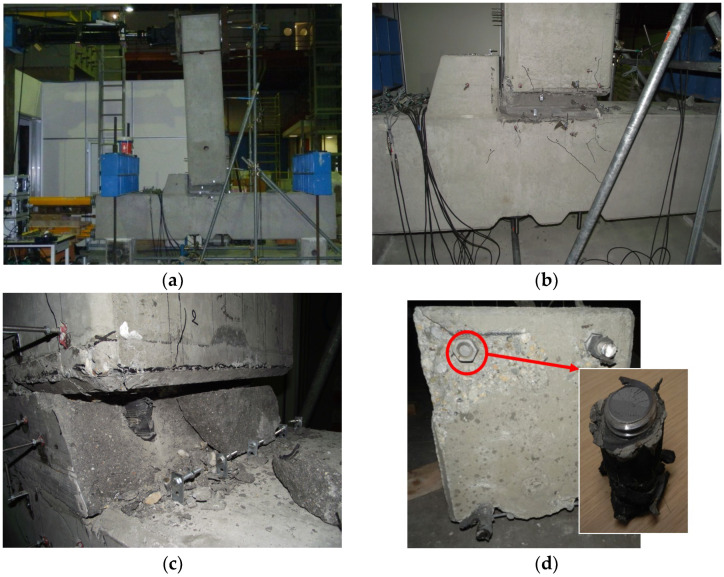
Experimental observations at advanced drift levels: (**a**) deformed shape of the beam at 3% drift, highlighting joint rotation and interface opening; (**b**) damage state at 3% drift, with visible cracking and grout detachment; (**c**) close-up view of the expelled and damaged concrete fragment at the beam–column interface; (**d**) fractured threaded bar segment observed at 4% drift, showing tensile rupture and residual bending.

Following this initial damage, the beam experienced a progressive deformation mechanism, characterized by a relative sliding motion between the beam and the column through the compromised grout interface. This mechanism subjected the threaded bars crossing the grout layer to a horizontal displacement of up to 20 mm, measured over a vertical length of approximately 200 mm. This critical region extended from the column–grout interface to a depth of around 100 mm inside the beam head. As a result, the bars developed a combined axial and bending deformation, with partial flexure occurring within the grout layer. This bending was exacerbated by the increasing crack width, which created eccentricities between the anchorage zone and the internal force path, promoting out-of-plane curvature of the bars. The phenomenon is clearly illustrated in Figure 8a, which shows the deformed shape of the beam at 3% drift, where the curvature and joint rotation become visibly pronounced.

Despite the progressive nature of the damage, the system maintained a stable hysteretic response up to a drift of 4%, beyond which a localized failure was observed Figure 8b,c. At this stage, one of the threaded bars fractured (Figure 8d) due to combined tension and plastic hinging, marking the first instance of structural strength degradation. Notably, even at this advanced damage level, global connection failure did not occur, demonstrating the inherent robustness of the hybrid connection system.

**Figure 9:**
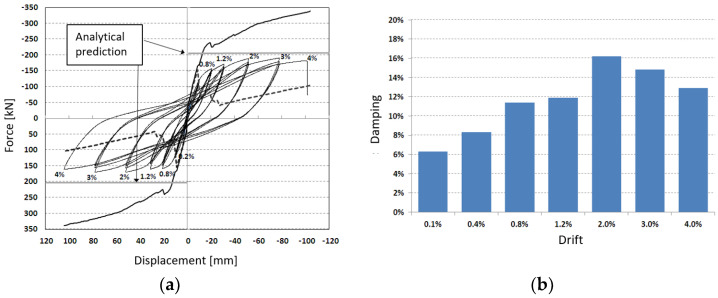
Experimental vs. numerical prediction (**a**) and damping values vs. drift level (**b**).

The force–displacement hysteresis loops (Figure 9a) obtained from the experimental test are presented and compared with the numerical predictions, which were developed under varying assumptions regarding interface degradation, bar flexure, and bond–slip behavior. While the model accurately reproduces the initial stiffness, it tends to overestimate the peak strength. This divergence is considered acceptable, as the numerical model was deliberately designed to be conservative, serving as a calibration tool for the experimental campaign rather than as a precise predictor of ultimate capacity. Despite this, the model remains effective in capturing the global deformation trends and the evolution of nonlinear response under increasing drift demands.

In addition, the equivalent viscous damping was evaluated for the initial cycles at each imposed drift level (Figure 9b). The results corrected using the Priestley–Calvi–Kowalsky formulation [76] demonstrated excellent energy dissipation capacity, with the damping ratio increasing steadily up to a drift level of 2%. This behavior reflects the activation of multiple dissipative mechanisms, including controlled interface sliding, bar deformation, and localized microcracking within the grout layer. Beyond 2% drift, a slight reduction in damping was observed, attributed to the onset of localized damage and partial degradation of the connection interfaces. Nevertheless, damping remained significant up to collapse, confirming the robust energy dissipation characteristics of the semi-rigid connection configuration even under large inelastic deformations.

To provide a quantitative measure of the connection’s seismic efficiency, normalized performance indices were derived from both experimental and numerical results. The ductility ratio (μ = Δu/Δy) reached approximately 5.0, indicating a deformation capacity significantly greater than the 3.0–3.5 range typically expected for precast RC connections designed to Eurocode 8. The equivalent viscous damping ($\xi_{\mathrm{eq}}$), computed from the enclosed hysteretic area and corrected following Priestley’s formulation, varied between 12% and 14% up to 3% drift, nearly three times higher than the 5% elastic reference adopted by EC8. Furthermore, the energy dissipation index ($E_{\mathrm{di}}$) showed that about 70% of the total energy input was dissipated through stable cyclic mechanisms, with limited stiffness and strength degradation. These results confirm that the proposed hybrid connection provides a quantifiable improvement in ductility and damping capacity compared with conventional pinned or semi-rigid precast joints.

The calibration of the numerical model was carried out through an iterative procedure, progressively adjusting the nonlinear properties of the joint components until the experimental and numerical responses converged. The comparison was evaluated both qualitatively, by matching the hysteretic shape, and quantitatively, through error metrics on key parameters. The peak load showed a difference of less than 8%, the initial stiffness within 10%, and the total dissipated energy up to 4% drift within 7%. These results confirm that, despite the simplifying assumptions (e.g., neglecting bar buckling and grout degradation), the model provides an accurate and robust representation of the joint’s global cyclic behavior.

The comparison between the experimental and numerical responses reveals an overestimation of strength in the analytical model. This difference is mainly attributed to the simplified representation of degradation phenomena such as bond–slip, grout cracking, and local stiffness loss, which were not explicitly included in the model to maintain computational efficiency and focus on the joint’s global cyclic behavior. These simplifications are consistent with the design-oriented objective of the study and with similar modeling approaches reported in the literature. Despite this, the correlation between experimental and numerical curves remains satisfactory, with close agreement in terms of initial overall stiffness degradation and shape of the initial response. The level of accuracy achieved is therefore considered appropriate for evaluating the global seismic performance and reliability of the proposed hybrid connection system.

## 6. Conclusions

This study presented an innovative connection solution for precast beam-to-column joints, following the Eucentre/DPC project [77], which is designed to enhance seismic performance while remaining compatible with dry construction practices. Through a combination of advanced numerical modeling and large-scale experimental testing, this work demonstrated the effectiveness of hybrid semi-rigid connections in delivering improved energy dissipation, displacement control, and structural robustness under cyclic seismic loading.

The global frame analysis, carried out using MIDAS GEN, highlighted the potential of the proposed connection strategy to reduce interstorey drift and limit second-order (P−Δ) effects. The introduction of partially restrained joints at interior supports, combined with hinged connections at external columns, resulted in a substantial increase in lateral stiffness and overall stability, while maintaining compatibility with common precast construction techniques.

A detailed finite element model of the beam-to-column joint, developed in MIDAS FEA, allowed for a realistic simulation of interface phenomena, such as grout–concrete detachment and localized bond–slip behavior. The model revealed the progressive development of stress concentrations and crack patterns, particularly in the grout layer and around the anchorage bars, anticipating the failure mechanisms later observed in the experimental phase.

The experimental results confirmed the transition from initial monolithic behavior to a semi-rigid joint response due to interface degradation. The system exhibited a stable and repeatable hysteretic response up to a drift of 4%, with no sudden strength loss, validating the ductility and energy dissipation potential of the proposed detail. The fracture of a single anchorage bar at peak drift did not trigger global collapse, underscoring the connection’s redundancy and damage-tolerant capacity. Moreover, the equivalent viscous damping increased up to a 2% drift and then slightly decreased, remaining at effective levels even at the highest deformation stages.

The equivalent viscous damping was derived from the area enclosed by the hysteretic loops of the initial stable cycles and subsequently corrected using the formulation proposed in [76]. This approach, widely adopted in performance-based seismic design, provides a consistent estimation of damping when complete cyclic degradation data are not available. Nevertheless, the equivalent viscous damping values presented here should be interpreted as indicative of the connection’s energy dissipation capacity rather than as generalized parameters. The quantitative correlation between local interface slips and global damping ratio requires repeated cyclic tests at different drift levels, which are planned for future research.

This hybrid connection concept offers a robust and economically viable alternative to external seismic isolation systems for precast buildings. It leverages familiar construction procedures while ensuring significantly improved post-earthquake performance, reduced downtime, and enhanced reparability. The results support a shift toward integrated, connection-based dissipation mechanisms within precast systems, aligning with performance-based seismic design principles.

It is acknowledged that the experimental campaign was limited to a single full-scale subassembly, which restricts the statistical reliability of the observed results. Consequently, no probabilistic inference or confidence level can be derived regarding the robustness or ductility parameters. The test should therefore be interpreted as a proof-of-concept validation of the proposed joint configuration, providing reference data for numerical modeling and identifying the governing load-transfer and energy-dissipation mechanisms. Future research will expand the investigation to a series of nominally identical specimens to capture the variability associated with workmanship, material dispersion, and assembly tolerances typical of precast construction.

Future developments will aim at generalizing the proposed detail for different precast typologies and scaling the concept for full structural frames. Further studies are also planned to refine the modeling of bar flexure and post-yield behavior and to explore cost-efficient grouting alternatives for widespread applications. Ultimately, this work contributes a practical and technically sound path for upgrading the seismic resilience of Europe’s precast industrial building stock.

## Figures and Tables

**Table 1:** Storey-level displacements and interstorey drifts from the nonlinear analysis of the proposed precast frame.

Story	Displacement [m]	Drift
3F	0.069	0.9%
2F	0.037	0.7%
1F	0.014	0.4%

**Table 2:** P−Δ stability checks under seismic loads at each storey.

Story	Storey Height [m]	Vertical Load [kN]	Story Shear Force [kN]	Story Drift [m]	Stability Coefficient (θ)	Allowable Limit	Remark	P−Δ
3F	3.5	4205.9	277.2	0.0284	0.1232	0.25	P−Δ Req.	1.1405
2F	3.5	11,377.2	386.2	0.022	0.1849	0.25	P−Δ Req.	1.2268
1F	4.0	19,587.6	597.2	0.0102	0.084	0.25	P−Δ Req.	1

**Table 3:** Drift levels applied to the beam during the quasi-static cyclic displacement history adopted for the experimental test.

Drift	
[%]	[mm]
0.1%	2.6
0.4%	10.4
0.8%	20.8
1.2%	31.2
2%	52
3%	78
4%	104

## Data Availability

The original contributions presented in this study are included in the article. Further inquiries can be directed to the corresponding author.
